# Experimental and Artificial Neuron Network Insights into the Removal of Organic Dyes from Wastewater Using a Clay/Gum Arabic Nanocomposite †

**DOI:** 10.3390/nano15110857

**Published:** 2025-06-03

**Authors:** Malak F. Alqahtani, Ismat H. Ali, Saifeldin M. Siddeeg, Fethi Maiz, Sawsan B. Eltahir, Saleh S. Alarfaji

**Affiliations:** 1 Department of Chemistry, College of Science, King Khalid University, Abha 61341, Saudi Arabia; angel.s.5@hotmail.com (M.F.A.); ssiddeeg@kku.edu.sa (S.M.S.); ssalarvagi@kku.edu.sa (S.S.A.); 2 Department of Physics, College of Science, King Khalid University, Abha 62529, Saudi Arabia; fmaiz@kku.edu.sa; 3 Department of Chemistry, College of Science, University of Hafr Al Batin, Hafr Al Batin 39524, Saudi Arabia; sawsanbt@uhb.edu.sa

**Keywords:** methylene blue, crystal violet, adsorption, clay, gum arabic, ANN

## Abstract

Organic dyes are pollutants that threaten aquatic life and human health. These dyes are used in various industries; therefore, recent research focuses on the problem of their removal from wastewater. The aim of this study is to examine the clay/gum arabic nanocomposite (CG/NC) as an adsorbent to adsorb methylene blue (MB) and crystal violet (CV) dyes from synthetic wastewater. The CG/NC was characterized using Fourier transform infrared spectroscopy (FTIR), X-ray diffraction (XRD), scanning electron microscopy (SEM), and Brunaure–Emmett–Teller (BET). The effect of parameters that may influence the efficiency of removing MB and CV dyes was studied (dosage of CG/NC, contact time, pH values, initial concentration, and temperature), and the optimal conditions for removal were determined. Furthermore, an artificial neural network (ANN) model was adopted in this study. The results indicated that the adsorption behavior adhered to the Langmuir model and conformed to pseudo-second-order kinetics. The results also indicated that the removal efficiency reached 99%, and q_max_ reached 66.7 mg/g and 52.9 mg/g for MB and CV, respectively. Results also proved that CG/NC can be reused up to four times with high efficiency. The ANN models proved effective in predicting the process of the removal, with low mean squared errors (MSE = 1.824 and 1.001) and high correlation coefficients (R^2^ = 0.945 and 0.952) for the MB and CV dyes, respectively.

## 1. Introduction

Water and wastewater treatment remain a major global challenge, particularly due to the presence of harmful organic dyes in industrial effluents. Methylene blue (MB) and crystal violet (CV) are widely used cationic dyes in textile and printing industries and are frequently detected in wastewater due to their high stability and resistance to biodegradation. Their toxicological effects on aquatic life and human health make them priority pollutants for adsorption studies [1,2].

Clay minerals, such as montmorillonite, kaolinite, and illite, are aluminosilicates known for their layered structures, high cation exchange capacity, and surface activity, making them effective adsorbents for cationic pollutants. Gum arabic, a natural polysaccharide, contains functional groups such as hydroxyl and carboxyl, which can participate in hydrogen bonding and weak electrostatic interactions. The combination of these materials is expected to yield a nanocomposite with enhanced adsorption performance due to the synergistic effect of the clay’s high surface area and the gum’s abundant active sites [3,4].

Various low-cost and natural adsorbents have been explored for removing methylene blue (MB) from wastewater. Materials like natural and purified clays [3], GA-based adsorbent [4], NiO/ZnO nanocomposites [5], wheat shells [6], biochar [7], and sugarcane [8] have shown promising MB removal efficiency, often following Langmuir isotherm and pseudo-second-order kinetic models. Some adsorbents, like biochar from municipal waste, need improved capacity to compete with more efficient alternatives. Clay-based adsorbents also showed strong performance [9], with high adsorption capacities and spontaneous nature, and often endothermic behavior. Magnetic nanocomposites (e.g., Fe_3_O_4_, chitosan-based, gum arabic-coated, and polyaniline–ferrite composites) [10,11,12] offer advantages in ease of separation and regeneration, although reusability sometimes reduces efficiency. Overall, many materials offer effective, low-cost alternatives to commercial adsorbents, though performance varies based on conditions like pH, temperature, and contact time.

Many previous studies have been reported that used different adsorbents to remove crystal violet (CV) dye from aqueous solutions. The powder of banana peels was utilized to decontaminate synthetic wastewater from CV. Results show that the adsorption process adheres to the pseudo-first-order and the Langmuir models. The removal efficiency reached only 93% [13]. Rice straw underwent thermochemical modification with citric acid to adsorb CV dye from water. The adsorption phenomenon was determined as both spontaneous and exothermic. The data fit a Langmuir isotherm and pseudo-second-order models [14]. Spent pepper seed powder (SSP) was used as a biosorbent to remove CV from wastewater. The adsorption process was characterized as spontaneous, physical, and endothermic. SSP was not reproduced and used for removal due to the cost of reproduction [15]. In another study, sawdust was used to adsorb CV from aqueous solutions. It was observed that solution pH was inversely proportional to the adsorption efficiency, and the process was exothermic [16]. Almond shell (AS) was also used to adsorb CV dye from aqueous solutions. The removal percentage was found to be only 83% [17]. In another study, NaOH-activated *Aerva javanica* leaf (NAJL) was examined as an adsorbent to remove CV dye from aqueous solutions. Although NAJL achieved maximum adsorption at 315.2 mg/g, it did not demonstrate good effectiveness when regenerated and reused [18]. Male flowers from the coconut tree were employed to activate carbon through the utilization of phosphoric and sulfuric acids (PAAC and SAAC, respectively). This activation process facilitated the adsorption of CV dye from aqueous solutions. The adsorption capacity of (PAAC) reached 60.4 mg/g, while (SAAC) reached 85.8 mg/g. However, the use of acids poses risks to both human health and the environment [19]. *Coniferous pinus* bark powder was used to remove CV dye. The maximum dye removal was about 8.9 mg/g, and the adsorption kinetics followed the pseudo-second-order. It is noted that the maximum adsorption capacity is low compared to that reported in other literature [20]. Bamboo chips modified with sodium carbonate showed spontaneous and endothermic adsorption with a capacity of 20.84 mg/g [21]. Activated carbon fibers achieved 91.2% removal in 20 min, though reuse was not evaluated [22]. A magnetite nanoparticle was used to remove CV from aqueous solutions with 20.9 mg/g capacity, but efficiency dropped to 63% after reuse [23]. Most of the previous studies lacked reuse evaluations, ANN modeling, or required longer contact time. Recent studies highlight the superior adsorption capacity of clay/gum arabic nanocomposites, attributed to their high surface area and synergistic interaction between components. These materials offer an eco-friendly and cost-effective alternative for the removal of organic dyes from wastewater.

Due to the cost and time consumption of experimental testing, adsorption processes are not always available for all operating conditions. Artificial intelligence can provide a powerful method to predict adsorption efficiency, such as using an artificial neural network (ANN) model [24].

Numerous studies have applied artificial neural networks (ANNs) to predict dye removal efficiency from aqueous solutions using various adsorbents. ANN models typically consist of input, hidden, and output layers, trained using algorithms like back-propagation or Levenberg-Marquardt, and are built using software such as Python, Maple, MATLAB, or JMP [25].

Different adsorbents, including natural materials, industrial by-products, and nanocomposites, have shown high dye removal efficiency using ANN models, often exceeding R^2^ = 0.95 and achieving low MSE/RMSE values, indicating strong predictive accuracy [26]. ANN inputs generally include parameters like pH, dye concentration, contact time, temperature, and adsorbent dosage. The majority of models demonstrated high accuracy in predicting the removal efficacy across different types of dyes. (e.g., CV, MB, eosin, malachite green, methyl violet, methyl orange, and Congo red), demonstrating the flexibility and reliability of ANNs in adsorption studies. Despite variations in data size and architecture (e.g., number of neurons and layers), the ANN approach consistently produced strong fits between predicted and experimental data, making it a valuable tool for modeling and optimizing dye adsorption processes [27].

Based on the preceding discussion, a clear research gap exists, as many studies report issues like long adsorption times, low capacities, high material costs, and limited testing of environmental impact, reusability, and high-temperature performance. Additionally, ANN modeling is often missing. Addressing these gaps can lead to more efficient and eco-friendly adsorption methods. This research introduces a novel, eco-friendly clay/gum arabic nanocomposite for removing MB and CV dyes, materials not previously used for this purpose. It also applies ANN modeling to predict and optimize removal efficiency. This study aims to synthesize and characterize a gum arabic/clay nanocomposite, evaluate its dye adsorption performance, analyze isotherm, kinetic, and thermodynamic behavior, and develop an ANN model to predict adsorption efficiency. The clay used in this study comprises montmorillonite, illite, kaolinite, quartz, and muscovite. These minerals provide a combination of high surface area (montmorillonite), ion exchange capacity (illite and montmorillonite), and structural stability (kaolinite and muscovite), which, when combined with the biopolymeric functionality of gum arabic, are expected to enhance the nanocomposite’s efficiency in adsorbing cationic dyes such as methylene blue and crystal violet.

## 2. Materials and Methods

Gum arabic was obtained from Nature Gums (Poole, UK), all other chemicals were purchased from Sigma-Aldrich (Burlington, MA, USA), and the natural clay was collected from Abha City, South of Saudia Arabia.

### 2.1. Synthesis of Clay/Gum Arabic Nanocomposite

A total of 25.0 g of gum arabic powder was combined with 50.0 mL of 99% pure ethanol. The mixture was allowed to sit overnight at 25.0 °C. To prepare the nanocomposite, 16.1 g of the ground clay was weighed and soaked in a flask containing 50 mL of ethanolic gum arabic solution. The mixture was stirred at approximately 25 °C for 24 h, then filtered to separate the solid contents. The solid contents were dried in an oven at about 105.0 °C for 3 h [28].

### 2.2. Batch Adsorption Experiments

In these experiments, varying concentrations (50 mg/L to 500 mg/L) of MB or CV solutions (50 mL) were placed in glass bottles, and then the desired amount of nanocomposite material (0.10 g per 50 mL of the adsorbate) was added to each bottle. The blend was stirred using a thermostated water bath shaker (200 rpm). Different factors, including the amount of adsorbent used, the duration of contact, the initial concentration, temperature (25.0 °C to 55.0 °C), and pH (2.0 to 11.0), were examined to understand their impacts. Each parameter was studied individually while keeping the other parameters constant. All experiments were conducted in triplicate to ensure reproducibility. The adsorbed amount was calculated via Equation (1) [29]:

(1) $$q_{e}=\frac{c_{o}-c_{e}}{M}\times V$$

where q_e_ (mg/g) is the dye uptake capacity, c_o_ and c_e_ (mg/L) are the initial and equilibrium concentrations of MB or CV, V is the volume of MB or CV solutions (L), and M is the CG/NC mass (g).

The removal efficacy was determined via Equation (2) [29]:

(2) $$R\%=\frac{c_{0}-c_{e}}{c_{0}}\times 100$$

The distribution coefficient (K_d_) of the dyes between the solid and aqueous phases is calculated using Equation (3) [29]:

(3) $$k_{d}=\frac{q_{e}}{C_{e}}$$

### 2.3. Kinetic Studies

Kinetic investigations were performed to analyze the adsorption behavior. The pseudo-first-order model is given in Equation (4) [29]:

(4) $$\ln\left(q_{e}-q_{t}\right)=\ln q_{e}-k_{1}t$$

where k_1_ is the adsorption rate constant, and t is the time.

The pseudo-second-order is determined using Equation (5) [30]:

(5) $$\frac{t}{q_{e}}=\frac{1}{k_{2}q_{e}^{2}}+\frac{t}{q_{e}}$$

where $k_{2}$ is the pseudo-second-order rate constant.

Intra-particle diffusion is presented in Equation (6) [30]:

(6) $$q_{t}=k_{id}t^{\frac{1}{2}}+I$$

$k_{id}$ signifies the diffusion rate constant (mg/g.min), while I is a constant indicating the width of the boundary layer (mg/g).

Elovich model is presented in Equation (7) [30]:

(7) $$q_{t}=\left(\frac{1}{\beta }\right)\ln\left(\alpha \beta \right)+\left(\frac{1}{\beta }\right)\ln t$$

where α and β are Elovich constants, and t is the time (min).

### 2.4. Adsorption Isotherm Models

Adsorption of MB or CV onto the surface of the CG/NC was studied by various adsorption isotherm models, viz. the Langmuir, Freundlich, and Temkin models, using Equations (8)–(10), respectively.

(8) $$\frac{c_{e}}{q_{e}}=\frac{1}{k_{L}q_{max}}+\frac{c_{e}}{q_{max}}$$

(9) $$\ln q_{e}=\ln k_{f}+\frac{1}{n}\ln C_{e}$$

(10) $$q_{e}=B\ln A+B\ln C_{e}$$

where q_max_ represents the maximum uptake capacity of CG/NC (mg/g), k_L_ is the Langmuir constant, and k_f_ represents the adsorption capacity of the sorbent. The n value determines the extent of non-linearity between the concentration of the solution and the adsorption process. A and B are Temkin isotherm constants, which were calculated from the intercept and slope of Equation (10).

### 2.5. Artificial Neural Networks (ANN)

**Figure 1:**
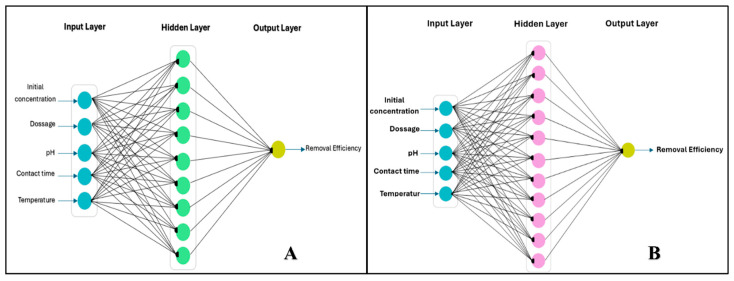
Schematic illustration of the ANN model used for predicting MB and CV dye removal efficiency. (**A**) MB; (**B**) CV.

A handmade algorithm was used to train the model based on gradient descent (CG) or Monte Carlo simulation methods. The algorithm is suitable if it achieves the lowest value of the mean squared error (MSE) and the highest value of the correlation coefficient (R^2^) by using Equations (1) and (2), and if the experimental data conform to the ANN-predicted data. The model was built on Maple 6 software (2024 version). The model consists of three layers: (i) the input layer, which contains five neurons for five parameters (initial concentration, dosage, pH, contact time, and temperature) for both dyes; (ii) the hidden layer, which contains one layer with nine neurons used for MB and eleven neurons for CV; and (iii) the output layer, which contains one neuron for the removal efficiency of the two dyes. A representative drawing of the model’s parts is shown in Figure 1.

### 2.6. Reusability of CG/NC

To regenerate the adsorbent, the CG/NC composite after adsorption was immersed in deionized water. The blend was continuously shaken at about 25 °C for a duration of 30 min. Afterward, the mixture was filtered, and the CG/NC was subsequently dried at 105 °C for 3 h [31].

### 2.7. Zeta Potential (ZP)

To evaluate the surface charge and zero-point values of the adsorbent, 25 mL of deionized water in eleven bottles was mixed with 0.20 g of the nanocomposite and stirred for ten minutes. To modify the mixture pH, NaOH or HCl solutions were used to adjust the pH within the range of 2.0 to 11.0. Then, the conductivity of each bottle was measured using a 4510 Conductivity-meter, both immediately and after 24 h [32].

### 2.8. Instrumentation

The MB and CV concentrations were measured before and after adsorption using Shimadzu UV/visible spectrophotometer (Shimadzu UV-1650, Kyoto, Japan). An attenuated total reflectance (ATR) spectroscopy instrument (Agilent Model Cary 630, Santa Clara, CA, USA) was utilized to identify the functional groups present in the samples. Spectra were recorded by the instrument within the range of 4000 cm^−1^ to 350 cm^−1^. A Shimadzu 6000 DX instrument diffractometer was used to investigate the prepared samples’ degree of crystallinity and morphological structure, where it was equipped with a graphite monochromator (CuKα, λ = 0.1541 nm). The technique measures the angles of diffraction and their intensities with a scan range of 2θ = 5–80° with steps of 0.02°. The SEM instrument used for this study was a JEOL JSM-7600f (Tokyo, Japan). Additionally, the SEM device was equipped with energy-dispersive X-ray (EDX) capabilities to determine the elemental ratio and quantify the amounts of elements present in the nanocomposite. The surface area (in m^2^/g) and pore size of the samples were evaluated using a Quantachrome Nova A4200E (Boynton Beach, FL, USA), at a temperature of 77 K.

## 3. Results and Discussion

### 3.1. Characterization

#### 3.1.1. Fourier Transform Infrared Spectroscopy (FTIR)

**Figure 2:**
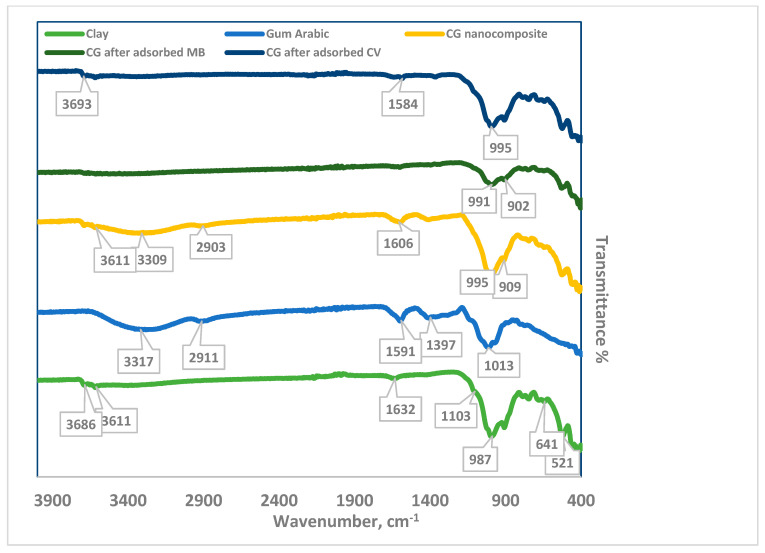
FTIR spectra of clay, gum arabic, CG nanocomposite, CG after adsorbing MB, and CG after adsorbing CV.

The functional groups of the clay, gum arabic, CG/NC, CG/NC after MB adsorption, and CG/NC after CV adsorption were identified by FTIR (Figure 2). The bands, around 3686 cm^−1^ and 3611 cm^−1^, suggest O-H stretching vibrations, indicating the presence of inner and outer structural hydroxyl groups or water molecules [33].

The band at 1632 cm^−1^ can be attributed to the H–O–H bending of absorbed water or bending vibrations of O–H groups in minerals within the clay structure. There are complex bands between 1400 cm^−1^ and 400 cm^−1^ in the fingerprint region due to overlapping vibrations [34]. In clays, Si–O stretching vibrations appeared at 1103 cm^−1^ for silicate minerals [34]. Al–OH bending vibrations can occur at 987 cm^−1^. The peaks at the range from 700–800 cm^−1^ revealed the deformation of Al–Al–OH and Mg–Al–OH, which indicate mixed-layer clays [35]. Si–O–Si and Si–O–Al bending vibrations are often observed around 641 cm^−1^ and 521 cm^−1^ in clays with aluminosilicate structures [34,35]. The broad peak at 3317 cm^−1^ belongs to O–H stretching vibrations in gum arabic [36]. The peak at 1591 cm^−1^ corresponds to the carbonyl group C=O [37]. The spectrum of CG/NC shows peaks at 3611 cm^−1^ and 3309 cm^−1^ for the hydroxyl group and O–H stretching vibrations. The aliphatic C–H stretching vibrations in gum arabic appear at 2903 cm^−1^. The peak at 1606 cm^−1^ corresponds to C=O stretching vibrations in gum arabic [33]. The spectrum also contains bending vibrations. The 900-800 cm^−1^ peaks were attributed to Si–O and Al–OH [35]. The FTIR spectrum of CG/NC after the adsorption of MB dye showed a weak peak around 1600 cm^−1^, which can be attributed to aromatic C=C stretching [38]. The peak at 991 cm^−1^ is commonly associated with out-of-plane C–H bending vibrations [39]. These observations suggest that the functional groups of CG/NC contribute to the adsorption of MB dye. In the case of CV adsorption, several characteristic peaks in the CG/NC spectrum either disappeared, shifted, or decreased in intensity—for example, the peak at 3309 cm^−1^. 

Additionally, the band around 1013 cm^−1^ can be attributed to the quaternary ammonium group (–N⁺(CH_3_)_3_). These spectral changes may indicate the formation of hydrogen bonds between CG/NC and the dye molecules or the presence of water [40].

#### 3.1.2. Scanning Electron Microscopy (SEM-EDX)

**Figure 3:**
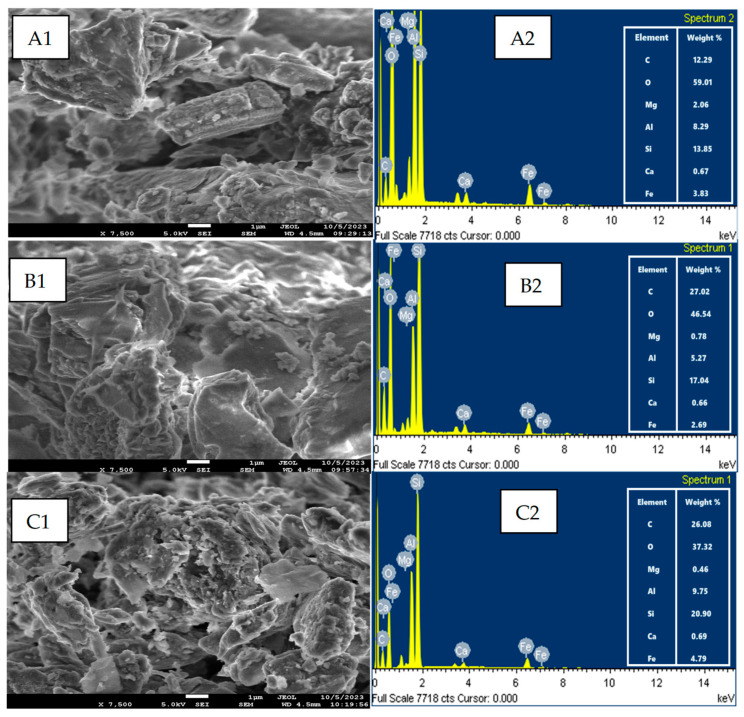
SEM-EDX analysis of CG/NC before and after adsorption of dyes. (**A1**,**A2**) SEM-EDX for CG/NC; (**B1**,**B2**) SEM-EDX for MB; (**C1**,**C2**) SEM-EDX for CV.

SEM analysis images of the CG/NC before and after the adsorption of MB and CV are displayed in Figure 3.

Prior to adsorption, the surface exhibited agglomerated structures with irregular morphology, as seen in Figure 3(A1). These structures are composed of compact clusters rather than clearly defined individual particles. After adsorption, the surface appeared noticeably rougher, with visible spots and surface irregularities. These changes are attributed to the accumulation of dye molecules on the CG/NC surface, as observed in Figure 3(B1) for MB and Figure 3(C1) for CV [31].

EDX analysis confirmed the existence of elements like O, C, Si, and Al in the CG/NC, with corresponding weight percentages listed in the inset table of Figure 2(A2). Following dye adsorption, the carbon content increased from 12.29% to 26.08% for MB and 27.02% for CV, as observed in Figure 3(B2,C2). These increases provide further evidence that adsorption happened primarily on the CG/NC surface [41,42].

#### 3.1.3. Brunauer–Emmett–Teller (BET) Analysis

The BET surface area measurements for clay, gum arabic, and the CG/NC nanocomposite are summarized in Table 1. Although a modest increase in surface area is observed upon composite formation, the absolute values remain relatively low. This can be attributed to the dense packing and agglomeration of clay particles, as well as the inherently non-porous nature of the gum arabic matrix, which limits nitrogen adsorption at cryogenic temperatures. Given these constraints, the determination of pore size and pore volume—particularly from the desorption branch of the nitrogen adsorption–desorption isotherm—is not considered reliable for materials with such low specific surface areas. Reporting these parameters under such conditions may lead to overinterpretation or misrepresentation of the material’s true porosity.

#### 3.1.4. X-Ray Powder Diffraction (XRD)

XRD was adopted to determine the crystallinity and identify the main minerals present in the samples.

**Figure 4:**
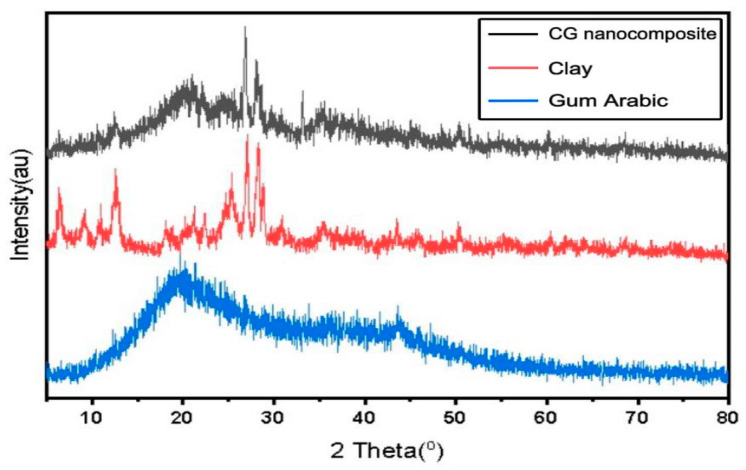
X-ray diffractogram of gum arabic, clay, and CG/NC.

Figure 4 shows the pattern displaying the distinguishing peaks of the clay at 6°, 9°, 25°, 27°, and 28° 2θ that are related to montmorillonite (001), illite (001), kaolinite (001), quartz (101), and muscovite (001), respectively [43]. XRD analysis indicated the gum arabic to be amorphous in nature, due to the absence of peaks in the diffraction pattern [44]. The formation of CG/NC led to the disappearance of some peaks and their intensities decreased due to the interaction between the gum arabic molecules and minerals in the clay. However, the presence of an amorphous peak next to some crystalline peaks provides further evidence of the formation of the compound [44,45].

To get the crystal size of the sample’s crystals, the Scherrer equation was used (Equation (11)). The average crystal structure sizes of the clay and CG/NC were found to be 39.70 nm and 27.75 nm, respectively.

D = kλ/β cos θ (11)

where D is the diameter of the crystallites (nm), β is half-peak width (FWHM) in radians, K is the Scherrer constant (0.9), λ is x-ray wavelength, and θ is the diffraction angle. The obtained findings conform to the previously reported results [46].

### 3.2. Artificial Neural Network (ANN)

The model was built to predict the removal efficacy of MB and CV dyes from aqueous solutions using a custom algorithm based on gradient descent (GD) and Monte Carlo simulation methods based on experimentally collected data. The experimental data used in the prediction process for MB and CV are listed in Tables S1 and S2. The predicted results were close to the experimental results, which proved its effectiveness in prediction. Its efficiency was also mathematically verified by finding the values (MSE = 1.824 and R^2^ = 0.945) and (MSE = 1.001 and R^2^ = 0.952) for the MB and CV, respectively.

### 3.3. Adsorption Studies

#### 3.3.1. Effect of Dosage of CG/MC

**Figure 5:**
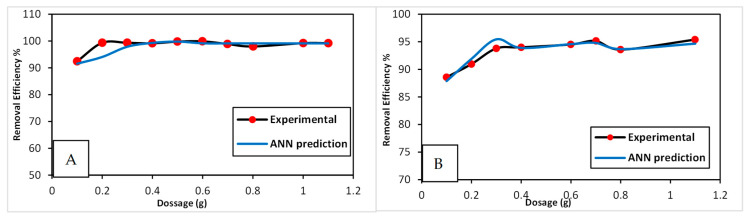
Removal efficiency for experimental and ANN-predicted data of MB and CV dyes vs. adsorbent dosage. (**A**) MB; (**B**) CV.

Figure 5 presents both the experimental and predicted ANN results. For MB dye (Figure 5A), increasing the CG/MC dose from 0.10 g to 0.20 g raised the removal efficiency gradually to 99.4%. Further increases in dosage showed no significant improvement in MB removal. For CV dye (Figure 5B), the removal efficacy increased from 88.6% to 95.4% as the dose rose from 0.10 g to 1.10 g, eventually reaching equilibrium. This trend is likely due to the increase in available active sites with higher adsorbent doses [31]. To maximize efficiency while minimizing adsorbent use, optimal CG/MC dosages of 0.20 g for MB and 0.30 g for CV were selected for the next experiments. The close agreement between experimental and ANN-predicted data confirms the model’s effectiveness in predicting dye removal efficiency.

#### 3.3.2. Effect of pH

**Figure 6:**
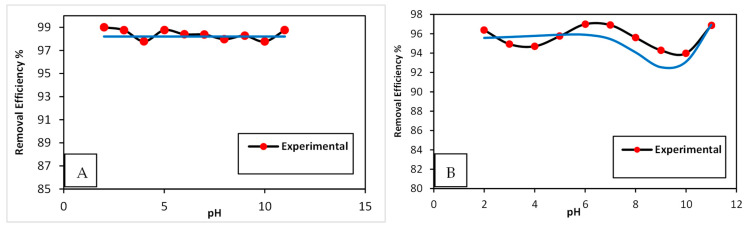
Removal efficiency for experimental and ANN-predicted data of MB and CV dyes vs. pH. (**A**) MB; (**B**) CV.

The change in pH is a significant factor that affects the accumulation of dyes on the adsorbent surface. Figure 6 shows the experimental data and ANN predicted for the removal efficacy of MB and CV dyes. It is obvious that the dyes were removed efficiently across the entire pH range (Figure 6). This could be due to the lack of repulsion between the neutral CG/NC surface and the cationic MB and CV dyes [31]. The normal pH value of the solution of 7.0 was used for the rest of the adsorption experiments for both dyes. The relative closeness between the experimental data and the predicted ANN demonstrates the success of the ANN models in predicting the removal efficiency of MB and CV dyes.

#### 3.3.3. Zeta Potential

**Figure 7:**
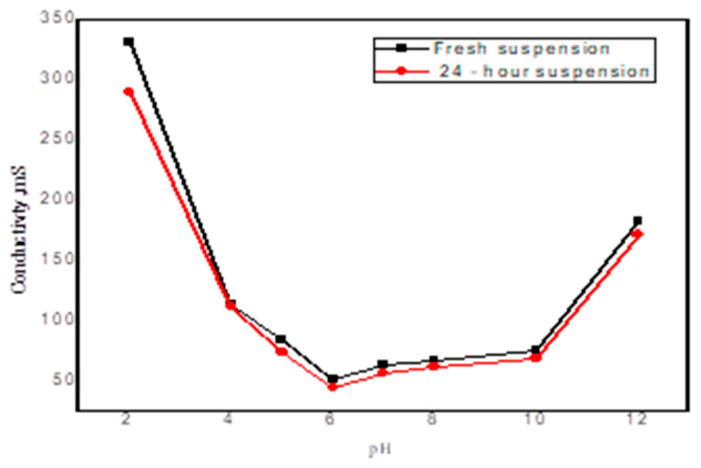
Surface charge density as a function of pH for CG/NC.

Zeta potential is a key parameter influencing adsorption through its effect on electrostatic interactions, adsorption kinetics, and competitive uptake among species. As illustrated in Figure 7, the CG/NC composite displays near-neutral zeta-potential values across the examined pH range. Although gum arabic and clay individually possess negative surface charges, due to acidic functional groups and aluminosilicate structures, the apparent neutrality of the composite may arise from strong agglomeration and surface shielding effects. These agglomerates can obscure the net surface charge by reducing the mobility and exposure of charged functional groups. Consequently, the negligible variation in dye removal efficiency with pH suggests that adsorption is not governed primarily by electrostatic interactions, but rather by non-electrostatic mechanisms such as hydrogen bonding, van der Waals forces, and physical adsorption.

#### 3.3.4. Effect of Contact Time

The impact of contact time was examined over a range of 10 s to 32 min, with other parameters held constant Figure S1 compares the experimental data with ANN predictions. Rapid adsorption of MB occurred within the initial minutes (Figure S1A), likely because of the availability of empty active sites on the CG/NC surface. After 18 min, the removal rate slightly declined, possibly because the adsorption sites were saturated. For CV dye (Figure S1B), 96.1% removal was achieved within two minutes, followed by equilibrium. This performance can be explained by the dye molecules occupying most of the active sites. The sorbent demonstrated a faster removal rate than previously reported materials, confirming its high efficiency [31]. Optimal contact times of 8 min for MB and 4 min for CV were selected for further studies. The close match between experimental and ANN-predicted data highlights the trustworthiness of the ANN models in estimating dye removal efficacy.

#### 3.3.5. Effect of Initial Concentration

The effect of varying the initial concentrations of the dyes from 50 mg/L to 500 mg/L was examined, with all other parameters held constant. Figure S2 illustrates the comparison between experimental data and predictions from the ANN models. The CG/NC demonstrated a high removal speed of MB dye at an initial concentration of 50 mg/L, achieving up to 99% removal (Figure S2A); however, as the initial concentration of MB increased, a slight decline in removal efficacy was observed. This decrease might be ascribed to the limited accessibility of active sites on the adsorbent and the intensified competition among MB molecules for these sites [9]. Conversely, the removal efficiency for CV dye slightly increased with higher initial concentrations of the CV solution (Figure S2B), possibly due to an enhanced driving force for molecular transfer [14]. An initial concentration of 50 mg/L was identified as optimal for conducting the adsorption experiments. The ANN models effectively predicted the removal efficiencies for both dyes, showing a strong correlation between the predicted and experimental data.

#### 3.3.6. Effect of Temperature

The impact of temperature on the adsorption efficiency of MB and CV dyes was investigated across a range from 25.0 °C to 55.0 °C while maintaining all other variables constant (Figure S3). For MB, an increase in removal efficiency was noted as the temperature rose, likely due to enhanced molecular collisions. Conversely, the efficiency of CV dye slightly decreased, potentially due to weaker interactions with the adsorbent. Room temperature (25.0 °C) was selected for subsequent experiments since temperature variations had a minimal effect. The experimental results aligned well with the predictions from artificial neural network (ANN) models, further validating the model’s accuracy.

### 3.4. Optimizing the ANN Model

To assess how well the ANN model could predict outcomes and to improve its reliability, an optimization algorithm was applied to estimate the removal efficiency of CG/NC under conditions that had not been tested in the lab. These predictions (Table 2) were then verified through follow-up laboratory experiments, which confirmed the model’s accuracy.

### 3.5. Thermodynamic Factors

**Figure 8:**
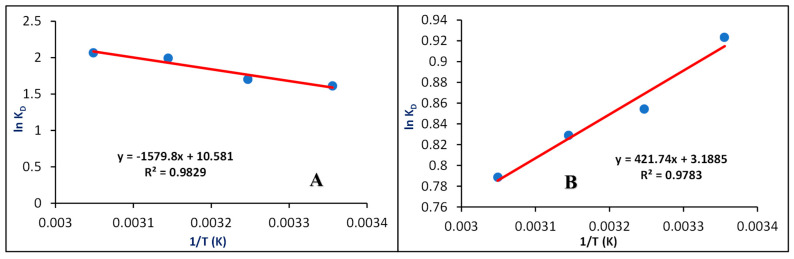
Thermodynamic factors for MB and CV dyes as a function of temperature. (**A**) MB; (**B**) CV.

The thermodynamic factors {enthalpy change (ΔH°), Gibbs free energy change (ΔG°), and entropy change (ΔS°)} are utilized to examine the nature of adsorption. Figure 8 shows a straight-line plot of ln K_D_ versus 1/T for the adsorption of MB and CV dyes onto the CG/NC. The ΔH° and ΔS° values are calculated using the slope and intercept of Equation (13), and ΔG° is calculated from Equation (12) [24].

(12) $$\Delta G=-RT\ln K_{D}\;$$

(13) $$\ln k_{D}=\frac{\Delta S^{0}}{R}-\frac{\Delta H^{0}}{RT}$$

where R represents the gas constant (8.314 J/k·mol), and T(K) denotes the temperature.

The positive value of ΔH° designates that the adsorption of MB dye is endothermic, whereas a negative ΔH° suggests that the adsorption of CV dye is exothermic. The positive ΔS° values for both dyes signify increased randomness within the adsorption system. The free energy change, ΔG°, for the adsorption of MB and CV on CG/NC was found to range from −3.991 to −5.631 kJ/mol for MB and −2.288 to −2.150 kJ/mol for CV, respectively. The negative values of ΔG° designate the spontaneous and physical nature of the adsorption process for both dyes. These results are consistent with previous reports, with reference [24,31]. The values of ΔG°, ΔS°, and ΔH° are detailed in Table 3.

### 3.6. Adsorption Isotherm Models

Isotherm models describe the interactions between the adsorbate and the adsorption sites [19]. In this work, several models were employed: Langmuir, Freundlich, and Temkin.

#### 3.6.1. Langmuir Isotherm Model

The Langmuir model for MB and CV molecules on the CG/NC is depicted in Figure S4. The mathematical expression for the Langmuir model is provided in Equation (4). Both dye adsorption systems follow the Langmuir model; this is evidenced by the high correlation coefficients (R^2^), demonstrating that the adsorption forms a monolayer on the adsorbent surface [24,30,31]. The maximum uptake capacities (q_max_) obtained were 66.7 mg/g for MB and 52.9 mg/g for CV. The adsorption nature is deemed favorable based on the separation factor (R_L_) calculated from Equation (4), which assesses the type of interaction between the dyes and CG/NC. R_L_ values show that the adsorption is unfavorable (R_L_ > 1), linear (R_L_ = 1), or favorable (0 < R_L_ < 1), with both dyes showing favorable adsorption [31]. The R_2_, q_max_, and K_L_ values for MB and CV are summarized in Table 4.

#### 3.6.2. Freundlich Isotherm Model

The Freundlich isotherm model was applied and is illustrated in Figure S5. The correlation coefficient (R^2^) for MB proposes that adsorption happens on a heterogeneous surface, as depicted in Figure S5A. The intercept and slope of the linear relationship were used to determine the values of K_f_ and n, which signify the adsorption capacity and the heterogeneity factor, respectively. The heterogeneity factor, n, designates the adsorption nature: linear if *n* = 1, chemical if *n* > 1, and physical if *n* < 1 [30]. For CG-MB, the n value is greater than 1, indicating that the adsorption process is chemical, which aligns with the reported value [24]. The values of R^2^, K_f_, and n for the adsorption of MB and CV dyes on the CG/NC are detailed in Table 4.

#### 3.6.3. Temkin Isotherm Model

The Temkin model posits that as the surface of the CG/NC becomes covered, the temperature of the molecules drops because of diminished interactions between the adsorbent and adsorbate. The constants A and B were calculated using the slope and intercept of Equation (10) and Figure S6. The correlation coefficients (R^2^), along with the constants A and B, are listed in Table 4. The relatively low Temkin B values (12.08 for MB and 28.55 for CV) indicate low to moderate interactions between the adsorbate and adsorbent, which is characteristic of physisorption. The high correlation coefficients (R^2^ = 0.929 for MB and 0.975 for CV) reflect a good fit to the Temkin model, suggesting a linear decrease in the heat of adsorption with increasing surface coverage. Additionally, the Temkin A constants (2.5 for MB and 2.6 for CV) support a moderate and uniform distribution of binding energies on the adsorbent surface.

**Table 4:** Parameters of the adsorption isotherm models.

Langmuir Isotherm			
	q_max_ (mg/g)	K_L_ (L/g)	**R^2^**
MB	66.7	0.159	0.991
CV	52.9	0.298	0.981
Freundlich Isotherm			
	N	K_f_ (mg/g)/(mg/L)	**R^2^**
MB	1.89	10.1	0.975
CV	2.76	16.5	0.880
Temkin Isotherm			
	A(L/g)	B	**R^2^**
MB	2.5	12.08	0.929
CV	2.6	28.55	0.975

### 3.7. Results of the Kinetic Studies

To study the adsorption kinetics, various kinetic models were investigated: pseudo-first-order, pseudo-second-order, intraparticle diffusion, and Elovich.

#### 3.7.1. The Pseudo-First-Order

The pseudo-first-order kinetic parameters for the adsorption of MB and CV are summarized in Table 5. The correlation coefficients (R^2^ values) for this model were 0.701 for MB and 0.323 for CV, indicating a poor fit to the experimental data. Additionally, the calculated equilibrium adsorption capacities (qₑ) were 5.694 mg/g for MB and 4.028 mg/g for CV, which appear to coincide numerically with those used in the pseudo-second-order model but should be interpreted cautiously given the low R^2^ values.

These results suggest that the pseudo-first-order model does not adequately describe the adsorption kinetics of either dye on the CG/NC composite. The low R^2^ values indicate that the assumption of adsorption being governed by a rate proportional to the number of unoccupied sites may not be valid in this system.

#### 3.7.2. The Pseudo-Second-Order

This model posits that the adsorption of the adsorbate onto the adsorbent surface involves a chemical process [30,31]. This model is distinguished by its ability to directly calculate the equilibrium adsorption capacity (qₑ) from the theoretical framework, rather than depending solely on experimental data. Figure S8 displays the plot of t/qₜ versus time (min), allowing for the calculation of the rate constant k_2_. The model precisely designated the adsorption kinetics for both dyes, evidenced by the high linearity of the plots (R^2^ = 0.997 for MB and R^2^ = 0.999 for CV). The qₑ values calculated from the model are closely aligned with those derived experimentally, affirming the model’s accuracy. These results conform with previous reports [30,31]. The values of R^2^, qₑ, and k_2_ for both dyes are summarized in Table 5.

#### 3.7.3. The Intra-Particle Diffusion Model

This model is used to analyze the migration of CV and MB dyes from wastewater to the CG/NC surface. This model, which helps understand adsorption kinetics within porous materials, considers factors like pore surface adsorption, film diffusion, surface diffusion, and pore diffusion as potential influences on the adsorption rate. However, the investigation, as depicted in Figure S9, revealed a noteworthy nonlinearity in the plot of q_t_ versus t^1/2^, with R^2^ values of 0.671 for MB and 0.511 for CV. This nonlinearity suggests that the intra-particle diffusion model does not suitably designate the adsorption kinetics of these dyes, indicating that the model’s assumptions may not align with the actual controlling mechanisms of dye adsorption [24]. The findings, detailed in Table 5, imply that intraparticle diffusion was not identified as the rate-controlling step, and boundary layer diffusion appears to play a negligible role in the adsorption process.

**Table 5:** Kinetic data of the adsorption of MB and CV dyes onto CG/NC.

Pseudo-First-Order			
	q_e_ (mg/g)	k_1_ (min^−1^)	**R^2^**
MB	5.694	0.000137	0.701
CV	4.028	0.00035	0.323
Pseudo-second-order			
	q_e_ (mg/g)	k_2_ (mg/g.min)	**R^2^**
MB	5.694	1.1876	0.997
CV	4.028	1.9537	0.999
Intra-particle diffusion			
	k_id_ (mg/g.min)	I	**R^2^**
MB	6.228	39.725	0.671
CV	4.111	12.827	0.511
Elovich			
	α	β	**R^2^**
MB	3.40 × 1029	10.98	0.704
CV	3.16 × 106	5.89	0.849

#### 3.7.4. The Elovich Model

This model postulates that adsorption rates are influenced by the obtainability of the active sites and the rate at which adsorbate molecules overcome the energy barrier for interaction. For MB, the empirical constants α and β were found to be exceptionally high at 3.40 × 10^29^ and 10.98, respectively, indicating a rapid initial adsorption rate and moderate adsorption capacity. Similarly, for CV, the values were α = 3.16 × 106 and β = 5.89, also suggesting quick adsorption kinetics and moderate capacity.

These values reflect the dynamics of how quickly adsorbate molecules interact with active sites and the extent of adsorption due to surface interactions and heterogeneity [24,31]. The values of α, β, and R^2^ for both dyes are obtained from Figure S10 and recorded in Table 5.

### 3.8. Results of the CG/NC Reusability 

**Figure 9:**
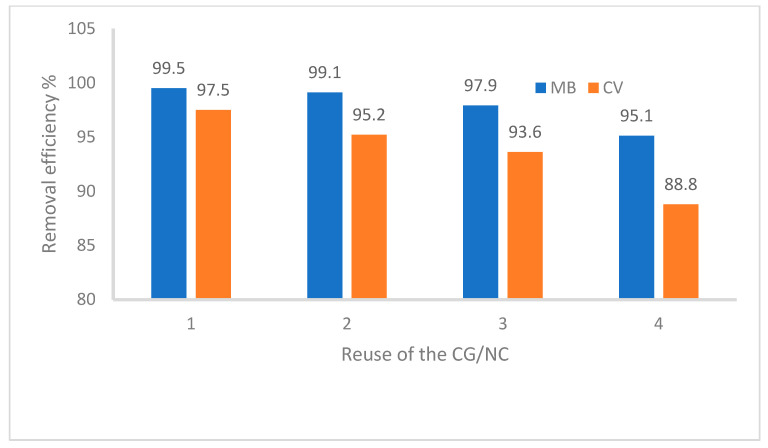
The reusability of CG/NC. 1–4 represent the reusability clycles.

The reusability of the CG/NC as an adsorbent is highlighted as a critical feature due to its ability to reduce costs, enhance environmental sustainability, conserve resources, and maintain consistent performance over time. The ease of dye removal by washing with water suggests that the adsorption is predominantly governed by physical interactions such as hydrogen bonding and electrostatic forces. Despite gum arabic’s known binding ability, no evidence of strong chemical bonding was observed in this system. Although the removal efficacy for MB and CV dyes decreases with each reuse, as shown in Figure 9, the CG nanocomposite remains effective for up to four cycles. This decline in efficiency is likely due to the structural degradation of some CG/NC molecules during the adsorption process. Despite this, the nanocomposite’s performance in repeated uses surpasses previously reported results, establishing it as a cost-effective and competitive option for dye removal.

### 3.9. Comparison with Other Adsorbents

Table 6 provides a comparative analysis of various adsorbents used to remove CV and MB dyes from wastewater. Most of these adsorbents follow the Langmuir isotherm and pseudo-second-order kinetic models, with optimal pH values typically ranging between 5.3 and 9.0. The data highlight the efficiency of CG/NC, which requires only 0.20 g for MB and 0.30 g for CV and operates optimally at a neutral pH of 7.0. This eliminates the necessity for chemical adjustment of pH, enhancing its practicality. In contrast, other adsorbents like PANI-NiFe_2_O_4_ and Khulays bentonite necessitate either acidic or alkaline conditions to function effectively. Thus, CG/NC is distinguished as one of the most effective and user-friendly adsorbents in this comparative study.

## 4. Conclusions

This study addressed the challenge of organic dye pollution through an economical and effective adsorption method. The CG/NC was synthesized and demonstrated an effective removal of MB and CV dyes from water. Among the factors tested, adsorbent dose was the most significant in enhancing removal efficiency. The adsorption process conformed to the Langmuir isotherm model, suggesting monolayer coverage of the dyes, and followed pseudo-second-order kinetics, with high accuracy shown by R^2^ values of 0.997 for MB and 0.999 for CV. Advanced ANN models employing gradient descent and Monte Carlo simulation techniques showcased robust predictive capabilities, evidenced by low MSE and high R^2^ values. The study confirms the potential of CG/NC as a low-cost, eco-friendly, and reusable solution for effectively mitigating dye contamination in water.

## Figures and Tables

**Table 1:** BET data for clay, gum arabic, and CG/NC.

Sample	Surface Area (m^2^/g)
Clay	14.34
Gum arabic	0.3835
CG/NC	46.91

**Table 2:** The optimization data for the removal efficiency of MB and CV dyes.

	Initial Concentration (mg/L)	Dosage CG/NC (g)	pH	Contact Time (min)	Temperature (°C)	R (%) Predicted	R (%) Experimentally
MB	50.00	0.30	7.00	7.00	24.00	99.15	97.31
CV	47.90	0.29	7.00	3.80	50.00	99.00	96.40

**Table 3:** Thermodynamic parameters of the adsorption of MB and CV dyes.

MB			
T, K	ΔG° (kJ/mol)	ΔS° (J/mol)	ΔH° (kJ/mol)
298	−3.991		
308	−4.356	88.01	13.13
318	−5.266		
328	−5.631		
CV			
T, K	ΔG° (kJ/mol)	ΔS° (J/mol)	ΔH° (kJ/mol)
298	−2.288		
308	−2.187	26.51	−3.50
318	−2.191		
328	−2.150		

**Table 6:** Comparison of optimal conditions and efficiency for different adsorbents.

Adsorbent	Adsorbate	Isotherm Model	Optimum pH	Kinetic Model	Enthalpy	q_max_ (mg/g)	Adsorbent Mass (g L^−1^)	Ref.
(RT)	MB	Langmuir	6–7	Second order	-	147	12.5	[47]
(Fe_3_O_4_/Mt)	MB	Langmuir	7.37	Second order	-	106.38	12.5	[11]
(IRKC)	MB	Langmuir-Freundlich	8	First order	-	240.4	10.0	[38]
PANI-NiFe_2_O_4_	MB	Langmuir	9	Second order	-	6.65	80.0	[10]
(WHS)	MB	Langmuir	7	Second order	endothermic	21.50	20.0	[6]
(LCF)	CV	Langmuir-Freundlich	7	First order	exothermic	34.12	5.0	[48]
Khulays natural bentonite	CV	Langmuir-Freundlich	5.3	Second order	endothermic	263	5.0	[49]
(NAJL)	CV	Langmuir	9	First order	exothermic	315.2	1.0	[18]
(OLP)	CV	Langmuir	7.5	Second order	-	181.1	4.0	[50]
(AS)	CV	Langmuir	6	Second order	endothermic	12.2	10.0	[17]
CG/NC	MB	Langmuir	7	Second order	endothermic	66.7	4.0	this study
CG/NC	CV	Langmuir	7	Second order	exothermic	52.9	6.0	this study

## Data Availability

The raw data supporting the conclusions of this article will be made available by the authors on request.

## Supplementary Materials

The supporting information can be downloaded at: https://www.mdpi.com/article/10.3390/nano15110857/s1, Table S1: Experimental conditions of MB removal efficiency for building an ANN; Table S2: Experimental conditions of CV removal efficiency for building an ANN; Figure S1: Removal efficiency for experimental and ANN-predicted data of MB and CV dyes vs. time. A denotes MB; B denotes CV; Figure S2: Removal efficiency for experimental and ANN-predicted data of MB and CV dyes vs. initial concentration. A denotes MB; B denotes CV; Figure S3: Removal efficiency for experimental and ANN-predicted data of MB and CV dyes vs. temperature. A denotes MB; B denotes CV; Figure S4: Linear fit of experimental data obtained using the Langmuir isotherm model. A denotes CG-MB; B denotes CG-CV; Figure S5: The Freundlich isotherm model. A denotes MB; B denotes CV; Figure S6: Linear fit of experimental data obtained using the Temkin isotherm model. A denotes CG-MB; B denotes CG-CV; Figure S7: Pseudo-first-order kinetic model for dye removal by CG/NC. A denotes MB; B denotes CG-CV; Figure S8: Pseudo-second-order kinetic model for dye removal by CG/NC. A denotes MB; B denotes CG-CV; Figure S9: Intra-particle diffusion kinetic model for dye removal by CG/NC. A denotes CG-MB; B denotes CG-CV; Figure S10: Elovich kinetic model for dye removal by CG/NC. A denotes CG-MB; B denotes CG-CV.

## References

[1] Jaramillo-Fierro X., González S., Jaramillo H.A., Medina F. (2020). Synthesis of the ZnTiO_3_/TiO_2_ Nanocomposite Supported in Ecuadorian Clays for the Adsorption and Photocatalytic Removal of Methylene Blue Dye. Nanomaterials.

[2] Ibupoto A.S., Qureshi U.A., Ahmed F., Khatri Z., Khatri M., Maqsood M., Brohi R.Z. (2018). Reusable carbon nanofibers for efficient removal of methylene blue from aqueous solution. Chem. Eng. Res. Des..

[3] Ouaddari H., Abbou B., Lebkiri I., Habsaoui A., Ouzzine M., Fath Allah R. (2024). Removal of Methylene Blue by adsorption onto natural and purified clays: Kinetic and thermodynamic study. Chem. Phys. Impact.

[4] Sandikci M.N., Isik B. (2025). Fabrication of calcium alginate/gum arabic/egg shell composite microbeads for adsorptive removal of methylene blue dye from aqueous solutions. Macromol. Res..

[5] Singh V., Sapehia R., Dhiman V. (2025). Removal of methylene blue dye by green synthesized NiO/ZnO nanocomposites. Inorg. Chem. Commun..

[6] Bulut Y., Aydın H. (2006). A kinetics and thermodynamics study of methylene blue adsorption on wheat shells. Desalination.

[7] Hoslett J., Ghazal H., Mohamad N., Jouhara H. (2020). Removal of methylene blue from aqueous solutions by biochar prepared from the pyrolysis of mixed municipal discarded material. Sci. Total Environ..

[8] Yang Z., Liu X., Liu X., Wu J., Zhu X., Bai Z., Yu Z. (2021). Preparation of β-cyclodextrin/graphene oxide and its adsorption properties for methylene blue. Colloids Surf. B Biointerfaces.

[9] Rehman M.U. (2021). Physicochemical characterization of Pakistani clay for adsorption of methylene blue: Kinetic, isotherm and thermodynamic study. Mater. Chem. Phys..

[10] Patil M.R., Shrivastava V. (2016). Adsorptive removal of methylene blue from aqueous solution by polyaniline-nickel ferrite nanocomposite: A kinetic approach. Desalination Water Treat..

[11] Chang J. (2016). Adsorption of methylene blue onto Fe_3_O_4_/activated montmorillonite nanocomposite. Appl. Clay Sci..

[12] Alzahrani E. (2014). Gum Arabic-coated magnetic nanoparticles for methylene blue removal. Int. J. Innov. Res. Sci. Eng. Technol..

[13] Azhar-ul-Haq M., Javed T., Abid M.A., Masood H.T., Muslim N. (2024). Adsorptive removal of hazardous crystal violet dye onto banana peel powder: Equilibrium, kinetic and thermodynamic studies. J. Dispers. Sci. Technol..

[14] Chowdhury S., Chakraborty S., Das P. (2013). Adsorption of crystal violet from aqueous solution by citric acid modified rice straw: Equilibrium, kinetics, and thermodynamics. Sep. Sci. Technol..

[15] Sulthana R., Taqui S.N., Syed U.T. (2022). RETRACTED: Adsorption of crystal violet dye from aqueous solution using industrial pepper seed spent: Equilibrium, thermodynamic, and kinetic studies. Adsorpt. Sci. Technol..

[16] Khattri S., Singh M. (2012). Use of Sagaun sawdust as an adsorbent for the removal of crystal violet dye from simulated wastewater. Environ. Prog. Sustain. Energy.

[17] Loulidi I., Boukhlifi F., Ouchabi M., Amar A. (2020). Adsorption of crystal violet onto an agricultural waste residue: Kinetics, isotherm, thermodynamics, and mechanism of adsorption. Sci. World J..

[18] AL-Shehri H.S., Almudaifer E., Alorabi A.Q., Alanazi H.S., Alkorbi A.S., Alharthi F.A. (2021). Effective adsorption of crystal violet from aqueous solutions with effective adsorbent: Equilibrium, mechanism studies and modeling analysis. Environ. Pollut. Bioavailab..

[19] Senthilkumaar S., Kalaamani P., Subburaam C. (2006). Liquid phase adsorption of crystal violet onto activated carbons derived from male flowers of coconut tree. J. Hazard. Mater..

[20] Ahmad R. (2009). Studies on adsorption of crystal violet dye from aqueous solution onto *Coniferous pinus* bark powder (CPBP). J. Hazard. Mater..

[21] Laskar N., Kumar U. (2018). Adsorption of crystal violet from wastewater by modified *Bambusa tulda*. KSCE J. Civ. Eng..

[22] Mulla B., Ioannou K., Kotanidis G., Ioannidis I., Constantinides G., Baker M., Hinder S., Mitterer C., Pashalidis I., Kostoglou N. (2024). Removal of Crystal Violet Dye from Aqueous Solutions through Adsorption onto Activated Carbon Fabrics. C.

[23] Ben Tahar L., Mogharbel R., Hameed Y., Noubigh A., Abualreish M.J.A., Alanazi A.H., Hatshan M.R. (2025). Enhanced removal of the crystal violet dye from aqueous medium using tripolyphosphate–functionalized Zn–substituted magnetite nanoparticles. Results Chem..

[24] Khan M.I., Almesfer M.K., Elkhaleefa A.M., Aamary A., Ali I.H., Shamim M.Z., Shoukry H. (2022). Efficient adsorption of hexavalent chromium ions onto novel ferrochrome slag/polyaniline nanocomposite: ANN modeling, isotherms, kinetics, and thermodynamic studies. Environ. Sci. Pollut. Res. Int..

[25] Abbasi E., Moghaddam M.R.A., Kowsari E. (2022). A systematic and critical review on development of machine learning based-ensemble models for prediction of adsorption process efficiency. J. Clean. Prod..

[26] Çelekli A., Geyik F. (2011). Artificial neural networks (ANN) approach for modeling of removal of Lanaset Red G on *Chara contraria*. Bioresour. Technol..

[27] Khonde R., Pandharipande S. (2012). Artificial Neural Network modeling for adsorption of dyes from aqueous solution using rice husk carbon. Int. J. Comput. Appl..

[28] Sharma K., Sharma S., Sharma V., Mishra P.K., Ekielski A., Sharma V., Kumar V. (2021). Methylene Blue Dye Adsorption from Wastewater Using Hydroxyapatite/Gold Nanocomposite: Kinetic and Thermodynamics Studies. Nanomaterials.

[29] Khan M.I., Yahya S.A., ElKhaleefa A., Shigidi I., Ali I.H., Rehan M., Pirzada A.M. (2025). Toxic Anionic Azo Dye Removal from Artificial Wastewater by Using Polyaniline/Clay Nanocomposite Adsorbent: Isotherm, Kinetics and Thermodynamic Study. Processes.

[30] Ali I.H., Bani-Fwaz M.Z., El-Zahhar A.A., Marzouki R., Jemmali M., Ebraheem S.M. (2021). Gum Arabic-Magnetite Nanocomposite as an Eco-Friendly Adsorbent for Removal of Lead(II) Ions from Aqueous Solutions: Equilibrium, Kinetic and Thermodynamic Studies. Separations.

[31] Elkhaleefa A., Ali I.H., Brima E.I., Shigidi I., Elhag A.B., Karama B. (2021). Evaluation of the Adsorption Efficiency on the Removal of Lead(II) Ions from Aqueous Solutions Using *Azadirachta indica* Leaves as an Adsorbent. Processes.

[32] Hussain S.A., Demirci Ş., Özbayoğlu G. (1996). Zeta potential measurements on three clays from Turkey and effects of clays on coal flotation. J. Colloid Interface Sci..

[33] Shehu Z. (2018). Synthesis, Characterization and Antibacterial Activity of Kaolin/Gum Arabic Nanocomposite on *Escherichia Coli* and *Pseudomonas Aeruginosa*. Res. J. Nanosci. Eng..

[34] Idrissi M. (2016). Degradation of crystal violet by heterogeneous Fenton-like reaction using Fe/Clay catalyst with H_2_O_2_. J. Mater. Environ. Sci..

[35] Adikary S., Wanasinghe D. (2012). Characterization of locally available Montmorillonite clay using FTIR technique. Annu. Trans. Inst. Eng. Sri Lanka.

[36] Upadhyay A. (2022). Ethylene scavenging film based on corn starch-gum acacia impregnated with sepiolite clay and its effect on quality of fresh broccoli florets. Food Biosci..

[37] Nayak A.K., Das B., Maji R. (2012). Calcium alginate/gum Arabic beads containing glibenclamide: Development and in vitro characterization. Int. J. Biol. Macromol..

[38] Jawad A.H., Abdulhameed A.S. (2020). Mesoporous Iraqi red kaolin clay as an efficient adsorbent for methylene blue dye: Adsorption kinetic, isotherm and mechanism study. Surf. Interfaces.

[39] Dai F., Zhuang Q., Huang G., Deng H., Zhang X. (2023). Infrared Spectrum Characteristics and Quantification of OH Groups in Coal. ACS Omega.

[40] Alorabi A.Q., Hassan M.S., Alam M.M., Zabin S.A., Alsenani N.I., Baghdadi N.E. (2021). Natural Clay as a Low-Cost Adsorbent for Crystal Violet Dye Removal and Antimicrobial Activity. Nanomaterials.

[41] Amirabadi S., Milani J.M., Sohbatzadeh F. (2020). Application of dielectric barrier discharge plasma to hydrophobically modification of gum arabic with enhanced surface properties. Food Hydrocoll..

[42] Mecheri R., Zobeidi A., Atia S., Neghmouche Nacer S., Salih A.A.M., Benaissa M., Ghernaout D., Arni S.A., Ghareba S., Elboughdiri N. (2023). Modeling and Optimizing the Crystal Violet Dye Adsorption on Kaolinite Mixed with Cellulose Waste Red Bean Peels: Insights into the Kinetic, Isothermal, Thermodynamic, and Mechanistic Study. Materials.

[43] Marsh A. (2019). Phase formation behaviour in alkali activation of clay mixtures. Appl. Clay Sci..

[44] Bashir M., Haripriya S. (2016). Assessment of physical and structural characteristics of almond gum. Int. J. Biol. Macromol..

[45] Emam H.E. (2019). Arabic gum as bio-synthesizer for Ag–Au bimetallic nanocomposite using seed-mediated growth technique and its biological efficacy. J. Polym. Environ..

[46] Yadav V.B., Gadi R., Kalra S. (2018). Synthesis and characterization of novel nanocomposite by using kaolinite and carbon nanotubes. Appl. Clay Sci..

[47] Nasuha N., Hameed B.H., Din A.T. (2010). Rejected tea as a potential low-cost adsorbent for the removal of methylene blue. J. Hazard. Mater..

[48] Ali R., Elsagan Z., AbdElhafez S. (2022). Lignin from agro-industrial waste to an efficient magnetic adsorbent for hazardous crystal violet removal. Molecules.

[49] Al-Shahrani S. (2020). Phenomena of removal of crystal violet from wastewater using Khulays natural bentonite. J. Chem..

[50] Elsherif K.M., El-Dali A., Alkarewi A.A., Ewlad-Ahmed A.M., Treban A. (2021). Adsorption of crystal violet dye onto olive leaves powder: Equilibrium and kinetic studies. Chem. Int..

