# Supplementary material for: Experimental and Artificial Neuron Network Insights into the Removal of Organic Dyes from Wastewater Using a Clay/Gum Arabic Nanocomposite
Source: Nanomaterials (Basel). 2025 Jun 3;15(11):857. doi: 10.3390/nano15110857 (PMC12157174; doi:10.3390/nano15110857)
Supplement: Supplementary file 1 [file nanomaterials-15-00857-s001.zip › nanomaterials-3610833-supplementary.pdf]

Table S1. Experimental conditions of MB removal efficiency for building an ANN.

Initial concentration (mg/L)	pH	Dosage (g)	Contact time (min)	Temperature (°C)	Removal efficiency (%)	Process parameters
50	7	0.1	30	25	88.58	Dosage
50	7	0.2	30	25	90.96	
50	7	0.3	30	25	93.80	
50	7	0.4	30	25	92.80	
50	7	0.6	30	25	92.52	
50	7	0.7	30	25	95.16	
50	7	0.8	30	25	93.58	
50	7	1.1	30	25	95.40	
50	2	0.3	30	25	97.38	pH
50	3	0.3	30	25	94.93	
50	4	0.3	30	25	94.40	
50	5	0.3	30	25	92.76	
50	6	0.3	30	25	96.99	
50	7	0.3	30	25	96.90	
50	8	0.3	30	25	95.60	
50	9	0.3	30	25	90.29	
50	10	0.3	30	25	93.98	
50	11	0.3	30	25	96.86	
50	7	0.3	0.17	25	74.37	Contact time
50	7	0.3	0.33	25	76.74	
50	7	0.3	1	25	86.49	
50	7	0.3	1.67	25	90.27	
50	7	0.3	2	25	96.07	
50	7	0.3	4	25	96.31	
50	7	0.3	6	25	96.24	
50	7	0.3	8	25	96.26	
50	7	0.3	10	25	95.34	
50	7	0.3	12	25	94.31	
50	7	0.3	14	25	93.36	
50	7	0.3	16	25	97.01	
50	7	0.3	18	25	96.20	
50	7	0.3	20	25	95.80	
50	7	0.3	22	25	96.23	
50	7	0.3	24	25	96.09	
50	7	0.3	26	25	95.83	
50	7	0.3	28	25	96.67	
50	7	0.3	30	25	96.23	
50	7	0.3	32	25	96.00	
50	7	0.3	4	25	94.13	Initial concentration
100	7	0.3	4	25	96.50	
150	7	0.3	4	25	97.42	
200	7	0.3	4	25	97.78	
250	7	0.3	4	25	99.07	
300	7	0.3	4	25	99.10	
350	7	0.3	4	25	98.88	
400	7	0.3	4	25	97.67	
450	7	0.3	4	25	97.98	
500	7	0.3	4	25	98.10	
50	7	0.3	4	25	96.79	Temperature

50	7	0.3	4	35	96.57	
50	7	0.3	4	45	96.49	
50	7	0.3	4	55	96.35	

Table S2. Experimental conditions of CV removal efficiency for building an ANN.

Initial concentration (mg/L)	pH	Dosage (g)	Contact time (min)	Temperature (°C)	Removal efficiency (%)	Process parameters
50	7	0.1	30	25	88.58	Dosage
50	7	0.2	30	25	90.96	
50	7	0.3	30	25	93.80	
50	7	0.4	30	25	92.80	
50	7	0.6	30	25	92.52	
50	7	0.7	30	25	95.16	
50	7	0.8	30	25	93.58	
50	7	1.1	30	25	95.40	
50	2	0.3	30	25	97.38	pH
50	3	0.3	30	25	94.93	
50	4	0.3	30	25	94.40	
50	5	0.3	30	25	92.76	
50	6	0.3	30	25	96.99	
50	7	0.3	30	25	96.90	
50	8	0.3	30	25	95.60	
50	9	0.3	30	25	90.29	
50	10	0.3	30	25	93.98	
50	11	0.3	30	25	96.86	
50	7	0.3	0.17	25	74.37	Contact time
50	7	0.3	0.33	25	76.74	
50	7	0.3	1	25	86.49	
50	7	0.3	1.67	25	90.27	
50	7	0.3	2	25	96.07	
50	7	0.3	4	25	96.31	
50	7	0.3	6	25	96.24	
50	7	0.3	8	25	96.26	
50	7	0.3	10	25	95.34	
50	7	0.3	12	25	94.31	
50	7	0.3	14	25	93.36	
50	7	0.3	16	25	97.01	
50	7	0.3	18	25	96.20	
50	7	0.3	20	25	95.80	
50	7	0.3	22	25	96.23	
50	7	0.3	24	25	96.09	
50	7	0.3	26	25	95.83	
50	7	0.3	28	25	96.67	
50	7	0.3	30	25	96.23	
50	7	0.3	32	25	96.00	
50	7	0.3	4	25	94.13	Initial concentration
100	7	0.3	4	25	96.50	
150	7	0.3	4	25	97.42	
200	7	0.3	4	25	97.78	

250	7	0.3	4	25	99.07	
300	7	0.3	4	25	99.10	
350	7	0.3	4	25	98.88	
400	7	0.3	4	25	97.67	
450	7	0.3	4	25	97.98	
500	7	0.3	4	25	98.10	
50	7	0.3	4	25	96.79	Temperature
50	7	0.3	4	35	96.57	
50	7	0.3	4	45	96.49	
50	7	0.3	4	55	96.35	

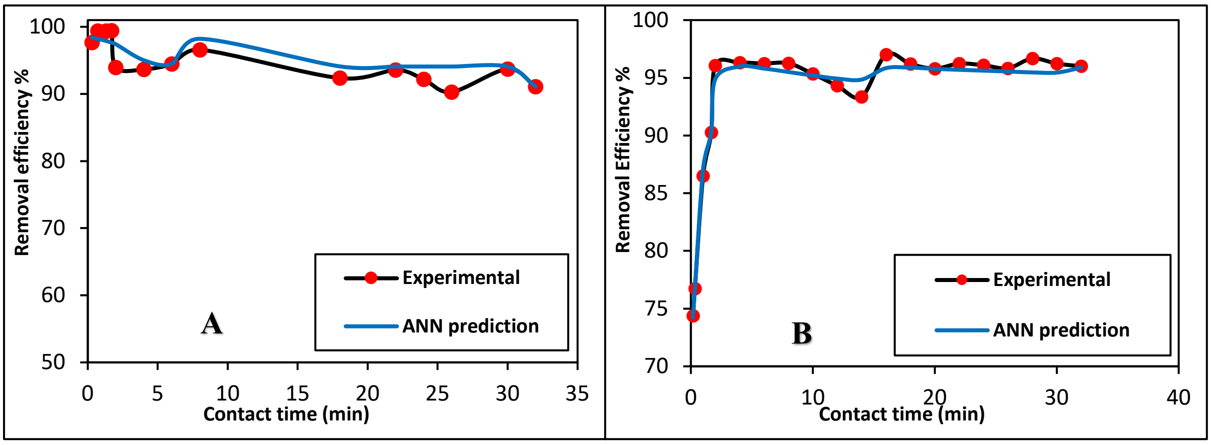

Figure S1. Removal efficiency for experimental and ANN-predicted data of MB and CV dyes vs. time. A denotes MB; B denotes CV.

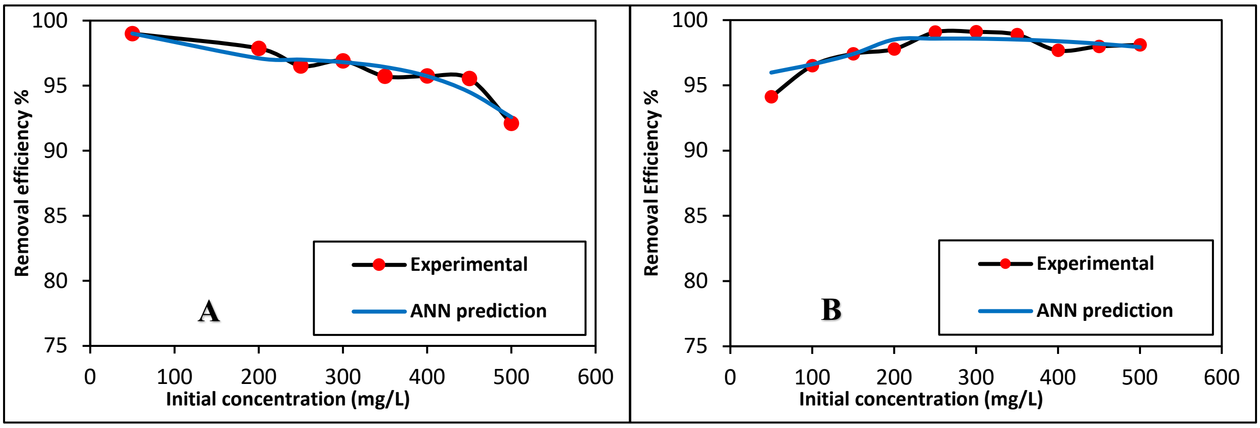

Figure S2. Removal efficiency for experimental and ANN-predicted data of MB and CV dyes vs. initial concentration. A denotes MB; B denotes CV.

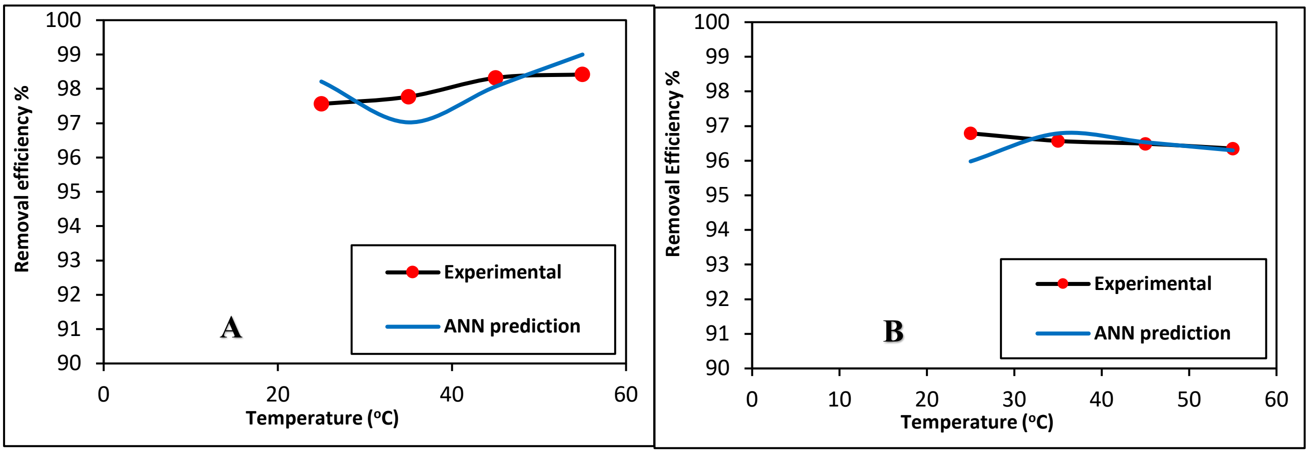

Figure S3. Removal efficiency for experimental and ANN-predicted data of MB and CV dyes vs. temperature. A denotes MB; B denotes CV.

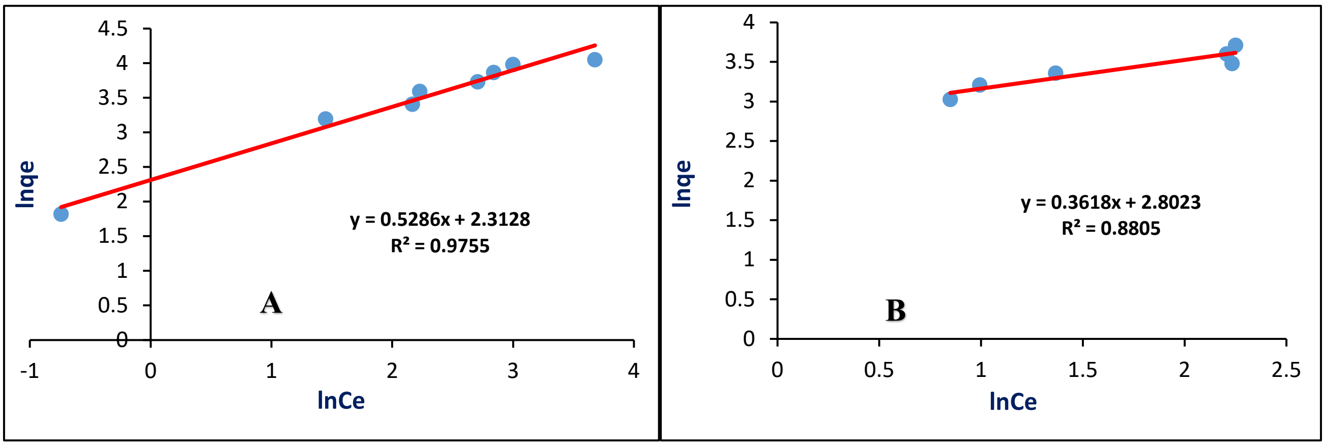

Figure S4. Linear fit of experimental data obtained using the Langmuir isotherm model. A denotes CG-MB; B denotes CG-CV.

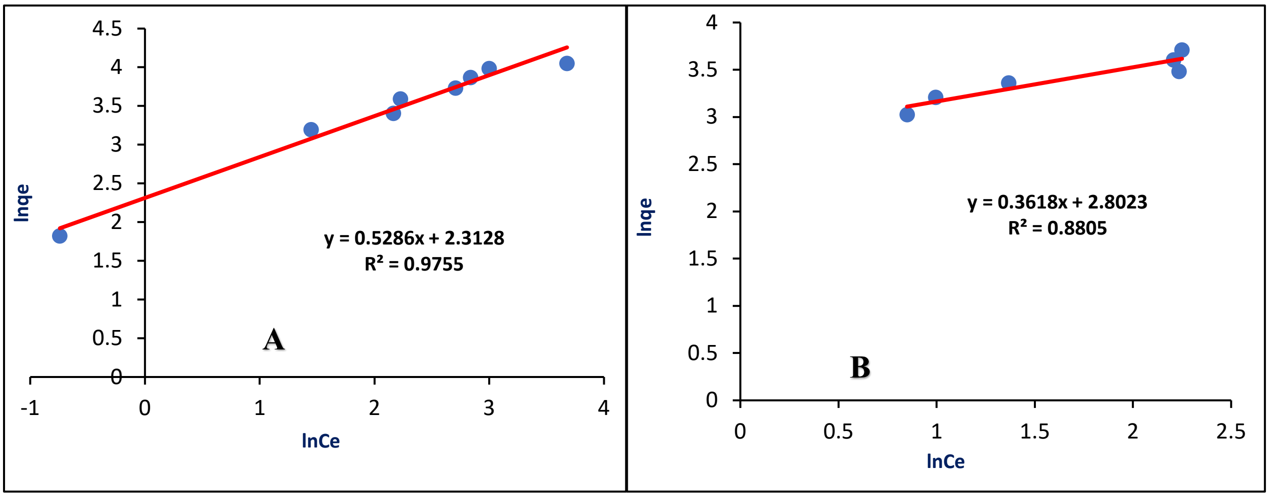

Figure S5. The Freundlich isotherm model. A denotes MB; B denotes CV.

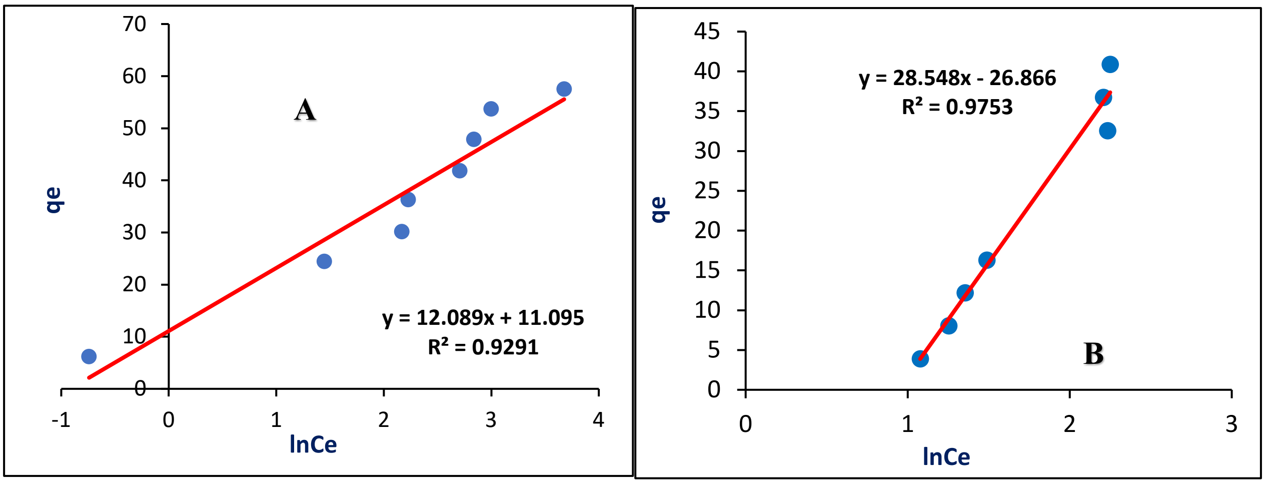

Figure S6. Linear fit of experimental data obtained using the Temkin isotherm model. A denotes CG-MB; B denotes CG-CV.

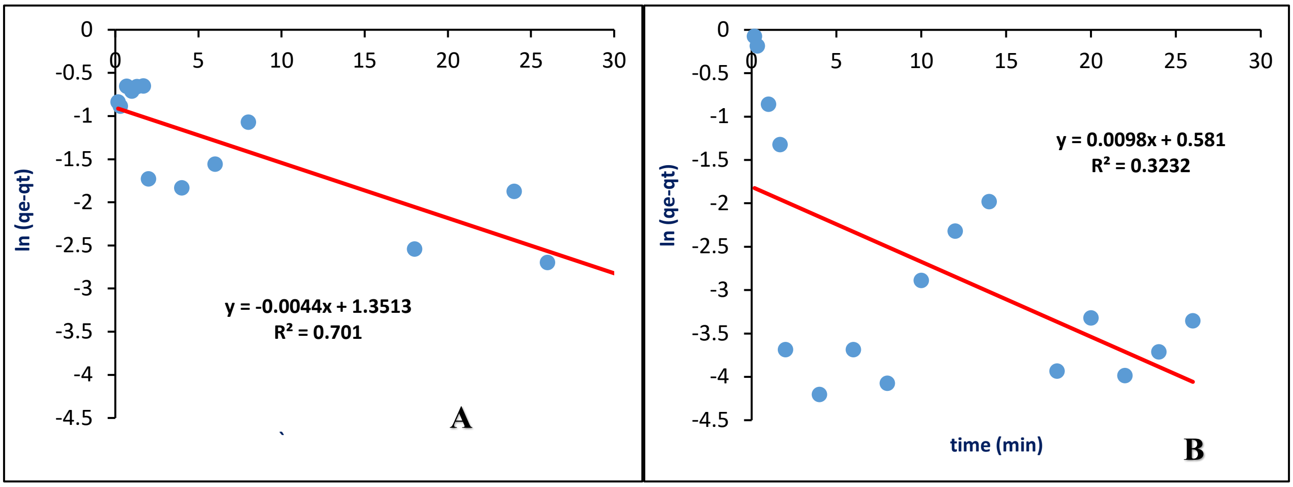

Figure S7. Pseudo-first-order kinetic model for dye removal by CG/NC. A denotes MB; B denotes CG-CV.

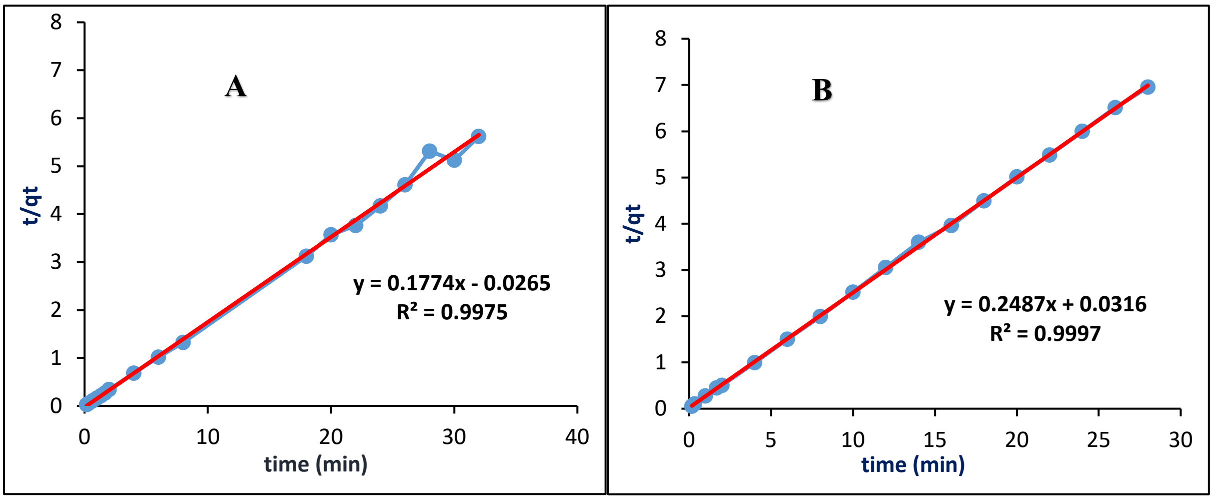

Figure S8. Pseudo-second-order kinetic model for dye removal by CG/NC. A denotes MB; B denotes CG-CV.

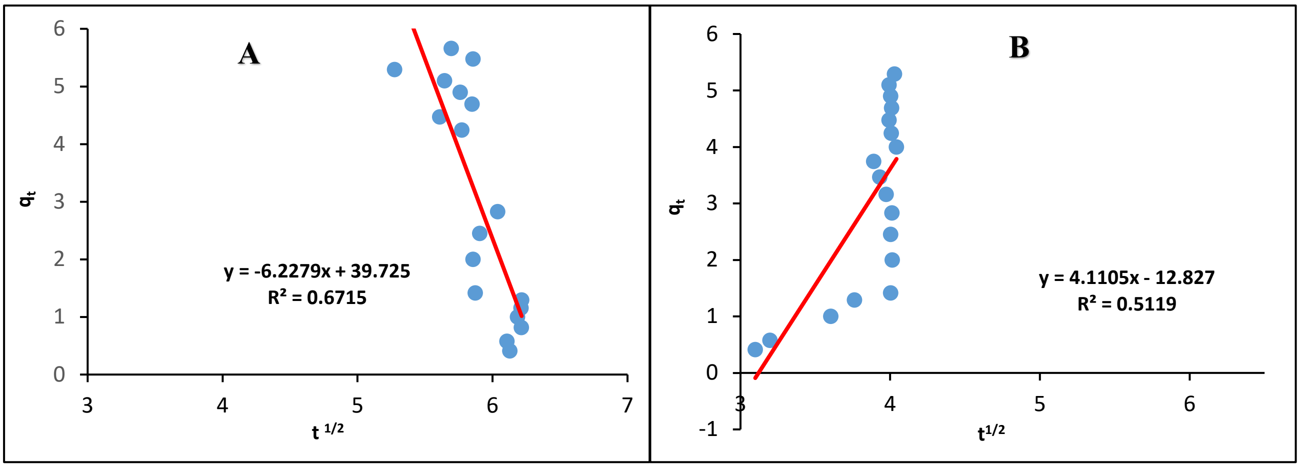

Figure S9. Intra-particle diffusion kinetic model for dye removal by CG/NC. A denotes CG-MB; B denotes CG-CV.

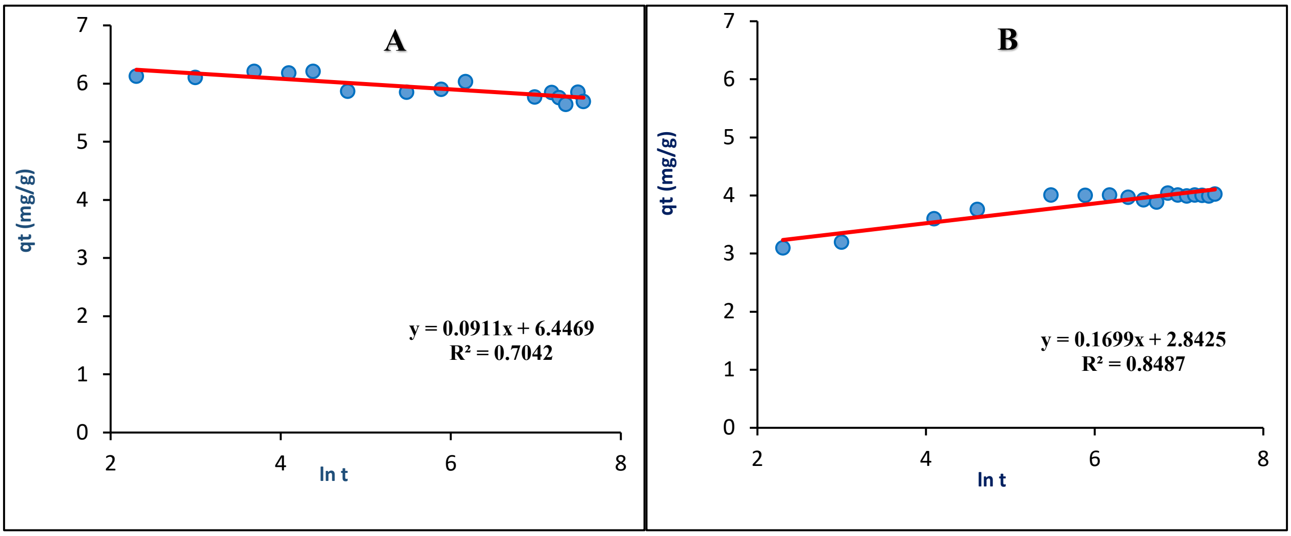

Figure S10. Elovich kinetic model for dye removal by CG/NC. A denotes CG-MB; B denotes CG-CV.
